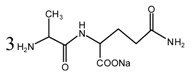# The Reaction between Furfuryl Alcohol and Model Compound of Protein

**DOI:** 10.3390/polym9120711

**Published:** 2017-12-14

**Authors:** Jiankun Liang, Zhigang Wu, Hong Lei, Xuedong Xi, Taohong Li, Guanben Du

**Affiliations:** 1 Yunnan Provincial Key Laboratory of Wood Adhesives and Glued Products, Southwest Forestry University, Kunming 650224, China; liangjiankun@swfu.edu.cn (J.L.); xuedongjx@163.com (X.X.); lith.cool@163.com (T.L.); 2 Material Science and Technology College, Beijing Forestry University, Beijing 100083, China; 3 College of Forestry, Guizhou University, Guiyang 550025, China; wzhigang9@163.com

**Keywords:** furfuryl alcohol, protein-based adhesives, model compound, modification mechanism

## Abstract

To guide the preparation of protein-based adhesive, especially the soy-based adhesive, the reaction between a simple dipeptide *N*-(2)-l-alanyl-l-glutamine (AG), being used as a model compound of protein, and its cross-linker furfuryl alcohol were studied in this paper. The products that were prepared with furfuryl alcohol and AG under different pHs were analyzed by ESI-MS, ^13^C NMR, and FT-IR. It was found that the medium environment had great effects on the competition of the co-condensation reaction between furfuryl alcohol and AG and self-condensation reaction of furfuryl alcohol molecules in the mixing system with furfuryl alcohol and AG. Under alkaline conditions, both co- and self-condensation were not obviously detected. Only when the value of pH was higher than 11, were a few co-condensation reaction products gotten. The reaction occurred mainly between furfuryl alcohol and the primary amido groups of AG. Under acid conditions, both co- and self-condensation were observed. The more acid the preparation conditions were, the easier to be observed the self-condensation of furfuryl alcohol molecules would be than the co-condensation between furfuryl alcohol and AG. When the value of pH was higher than 5, both co- and self-condensation were not outstanding. In this study, under pH 3, the co- and self-condensation found equilibrium. There was a great possibility for the primary amido and aliphatic amino groups of AG molecules to react with furfuryl alcohol molecules. No reaction was detected between the secondary amido groups of AG and furfuryl alcohol.

## 1. Introduction

The application of soy protein-based adhesives in wood industry has been reported [1,2,3]. To meet with the requirements on mechanical performances and water resistance, soy protein-based adhesives normally has to be modified with some cross-linkers, such as formaldehyde and its derivatives, isocyanate, epoxy, etc. [4,5,6,7,8,9]. The modification mechanism was presumed to be based on the reaction between the cross-linkers and the reactive groups from protein molecules, which including –NH_2_, –CO–NH–, –CO–NH_2_, –COOH, –OH, –SH, and –Ph–OH [10,11,12].

Because of the similar chemical structure of furfuryl alcohol as formaldehyde and its derivatives, furfuryl alcohol can be used as a cross-linker of protein-based adhesives, which has already been proved by the study of Kumar [13,14]. Furfuryl alcohol is the hydrogenation product of furfural, which can be gotten from some bio-based materials, such as corn, wheat, etc. Being different from petroleum-based formaldehyde and its derivatives, furfuryl alcohol is sustainable and environment-friendly, which are definitely favorable for being a cross-linker of protein-based adhesives [15,16]. After all, the toxicity caused by the addition of formaldehyde and its derivatives are not desirable for the usage of protein-based adhesives, although the addition amount of the cross-linker might be low.

Under determined conditions, furfuryl alcohol molecules have a tendency of self-condensation to get furfuryl alcohol resin. With its good mechanical performances and heat and water resistance, the resin can be used as adhesives of wood, rubber, metal, and ceramic [17,18,19]. When using as a cross-linker of protein-based adhesives, the competition between the self-condensation of furfuryl alcohol and the co-condensation of furfuryl alcohol and protein has to be considered.

Since the composition amino acids units of protein were very complex and the soy flour for the preparation of soy protein-based adhesives was a mixture with some ingredients, it seems almost impossible to study the modification mechamisms with a soy flour material. Based-on our previous study [20,21,22], *N*-(2)-l-alanyl-l-glutamine (AG) was chosen as a model compound to react with furfuryl alcohol to simplify the study. The objective of this study is to find suitable reaction condition when using furfuryl alcohol to cross-link the protein molecules, and then be guidance on the preparation of soy protein-based adhesives. The main reason for the choosing of AG is that it is an easily-gotten dipeptide. Although it cannot represent soy protein, its reaction with furfuryl alcohol can still give some useful information on the reaction pH and the possible reactive groups of amino acid, dipeptide or other compositions of protein molecules when using furfuryl alcohol as a cross-linker of protein-based adhesives. After all, AG owns some similar characteristic groups as those of soy protein, specifically, –NH_2_, –CO–NH–, –CO–NH_2_, and –COOH. The mechanism on the modification of protein-based adhesives is rather few till now. The study on the reaction with AG model compound and furfuryl alcohol will be just a beginning to pursuing the law for the modification of soy protein-based adhesives and even other protein-based ones.

## 2. Materials and Methods

### 2.1. Materials

*N*-(2)-l-alanyl-l-glutamine with a purity of 99%, furfuryl alcohol (FA, 98.5 wt %), toluene-p-sulfonic acid (99 wt %) and sodium hydroxide (96 wt %) in reagent grade were purchased from Sinopharm Chemical Reagent Co., Ltd., Beijing, China.

### 2.2. Preparation of Resins

When considering the pH affect the reactivity of furfuryl alcohol and protein molecules, some samples with AG and furfuryl alcohol were prepared under different pH conditions. The objective was to see whether the co-condensation of AG and furfuryl alcohol exists besides the self-condensation of furfuryl alcohol in the mixing system and what were the favorable conditions for the reactions. The preparation procedure was as below: The AG and furfuryl alcohol with molar ratio n(FA)/n(AG) = 3:1 were charged to a flask equipped with a condenser, thermometer and a magnetic stirrer bar. Some distilled water was added to get a mixture with solid content 30%. The mixture was then kept at 75–80 °C for 1 h under a desired pH. The pH was adjusted with toluene-p-sulfonic acid saturated solution or 20% sodium hydroxide solution and was kept as 1, 3, 5, 7, 9, 11, and 13, respectively, to get a series of samples with the name of FA/AG.

To see the effects of pH on the self-condensation of furfuryl alcohol, two samples with furfuryl alcohol materials were prepared under two different pHs. The preparation procedure was as below: some furfuryl alcohol and distilled water were charged to a flask equipped with a condenser, thermometer, and a magnetic stirrer bar to get a mixture with solid content 30%. Toluene-p-sulfonic acid saturated solution or 20% sodium hydroxide solution was used to adjust the pH. The mixture being kept at 75–80 °C for 1 h at pH 1 was named as FA1 and that at pH 11 was named as FA2.

### 2.3. Electrospray Ionization Mass Spectrometry (ESI-MS)

The spectra were recorded on a Waters Xevo TQ-S instrument. The FA/AG and FA series samples were dissolved in chloroform, respectively, at a concentration of about 10 μL/mL and then were injected into the ESI source plus ion trap mass spectrometer via a syringe at a flow rate of 5 μg/s. Spectra were recorded in a positive mode, with ion energy of 0.3 eV and scan range of 0–1000 Da.

### 2.4. ^13^C NMR

The 300 μL liquid sample was directly mixed with 100 μL DMSO-d6 for ^13^C NMR determination. The spectra were obtained on a Bruker AVANCE 600 NMR spectrometer using 12 μs pulse width (90°). The relaxation delay was 6 s. To achieve a sufficient signal-to-noise ratio, inverse-gated proton decoupling method was applied. The spectra were taken at 150 MHz with 800–1200 scans accumulated.

### 2.5. FT-IR Analysis

The KBr pills with AG material and FA/AG series samples were prepared for FT-IR analysis. The solid AG material was pre-dried to a constant weight. The liquid FA/AG series samples were freeze-dried in advance to remove the water, too. The FT-IR spectra were gotten on a Varian 1000 infrared spectrophotometer.

## 3. Results and Discussion

### 3.1. The ESI-MS Analysis

**Figure 1:**
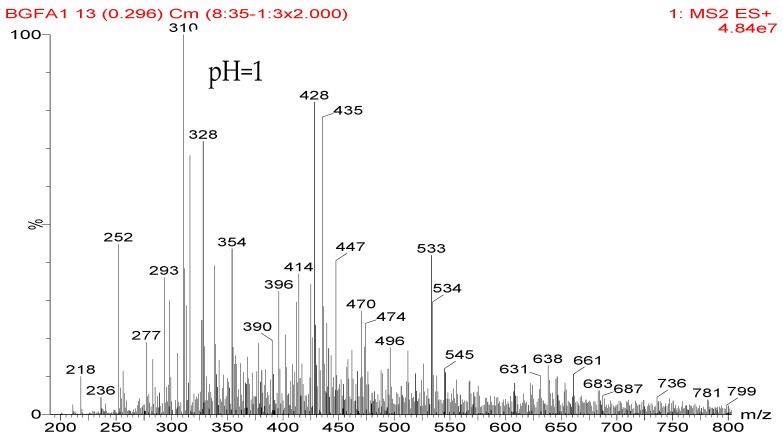

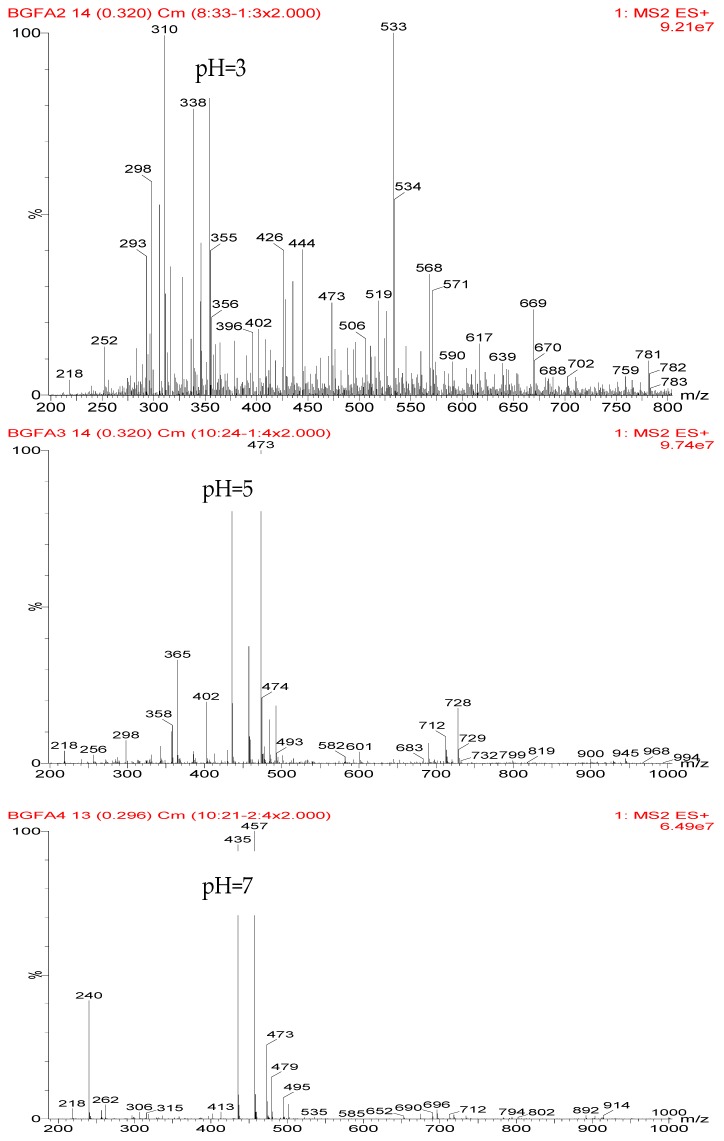

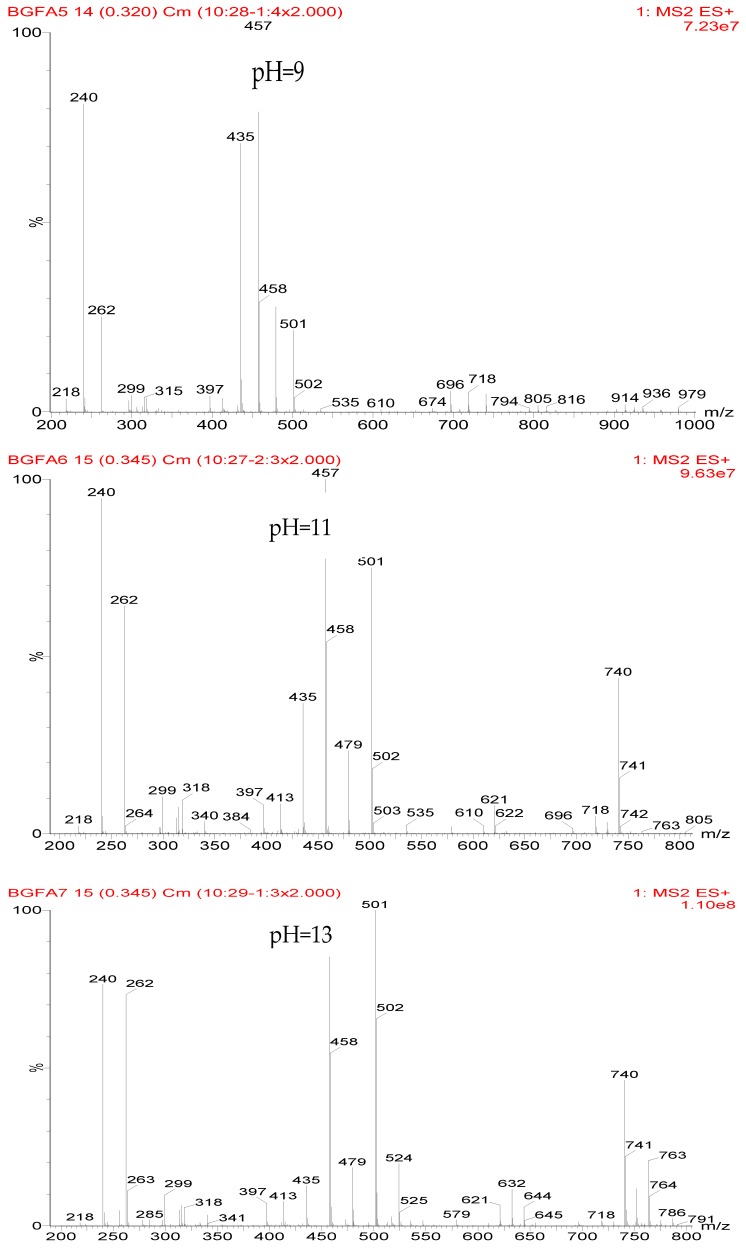
ESI-MS spectra of the samples with furfuryl alcohol and *N*-(2)-l-alanyl-l-glutamine prepared under different pHs.

The ESI-MS spectra of FA/AG series samples prepared at different pHs, specifically pH = 1, 3, 5, 7, 9, 11, and 13, are given as Figure 1. The assignments on the main ion peaks gotten under acid and alkaline conditions are given in Table 1 and Table 2, seperately. According to the mechanism of ESI-MS and the chemical structure of furfuryl alcohol and AG molecules, the ion peaks observed in the ESI-MS spectra mainly came from ions in three forms, namely [M + H]^+^, [M + Na]^+^, and [M + K]^+^. The former came from the combination of amino groups in AG molecules and H^+^ and the latter two came from the combination of oxygen atom from carbonyl groups in AG molecules and Na^+^ or K^+^. Since there were p–π conjugated structure in furfuryl alcolhol molecules, the furfural carbonium ions were possible to be detected.

In Figure 1, more ion peaks could be detected for FA/AG series samples prepared under acid conditions than those prepared under alkaline conditions. In theory, hydroxymethyl furfural carbonium ions might be the active intermediates. Under acid conditions, with a higher H^+^ concentration, the concentration of the carbonium ions would increase and then be helpful for the reaction between furfuryl alcohol and AG. While under alkaline conditions, the furfural carbenium ions become unstable [23].

According to the calculation of the ion peaks, the peak of 218 Da came from the AG molecules, which meant the existence of the free AG. Although the intensity from this peak was not high, it could be detected in all of the samples. In this work, the acid conditions were adjusted by toluene-p-sulfonic acid with molecule weight 172 Da. The ions with 293 and 533 Da from the samples prepared under acid conditions were then be assigned as the combination of the toluene-p-sulfonic acid and FA or their self-condensates.

During the self-condensation of furfuryl alcohol molecules, formaldehyde might be given off for the relatively unstable methylene ether link, seen as below:


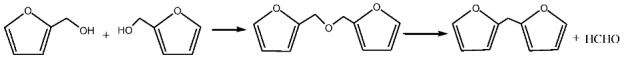


In Table 1, the peak of 298 Da was assigned as the co-condensation product of furfuryl alcohol and AG and that of 617 Da came from the Na^+^ complex with two molecules of the products. Seen from Figure 1, the ions with 298 Da could be detected clearly for the samples prepared under pH 1 and 3. Then, the intensity of the ion peak decreased for the sample prepared under pH 5. Even no peak at 298 Da could be detected for the sample prepared under pH 7. Although the peak at around 298 Da reappeared for the samples prepared under pH 9, 11, and 13, judged by the intensity of this peak, the reaction between furfuryl alcohol and AG under alkaline conditions was not as easy as that under acid conditions. The peaks of 252 and 310 Da could be assigned as carbonium ions that were gotten from the co-condensation product of furfuryl alcohol and AG. The possible reaction for the formation of the carbonium ions of 310 Da was as below:


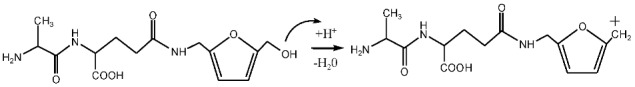


Table 2 was the assignments on the main ion peaks of the samples with furfuryl alcohol and AG prepared under alkaline conditions. It was regretful that there was no co-condensate with furfuryl alcohol and AG was detected. The peaks of 218, 240, and 256 Da came from one molecule of AG. The peaks of 435, 457, and 479 Da came from the complex of two molecules of AG with H^+^. The peaks of 501, 718, and 740 Da were from the complex of two molecules of AG with Na^+^. The peaks of 299, 315, 318, 397, and 413 Da were from furfuryl alcohol and its derivatives.

In all, the analysis on the ESI-MS spectra indicated that the co-condensation occured only under acidic conditions. However, since one AG molecule owns three different amino groups, specifically, primary amido, secondary amido, and aliphatic amino groups, and all of them have the possibility to react with furfuryl alcohol in theory, the analysis of the mechanism of the reaction between AG and furfuryl alcohol is still impossible with the limited information that is given by the ESI-MS results with so many isomers.

### 3.2. The ^13^C NMR Analysis

**Figure 2:**
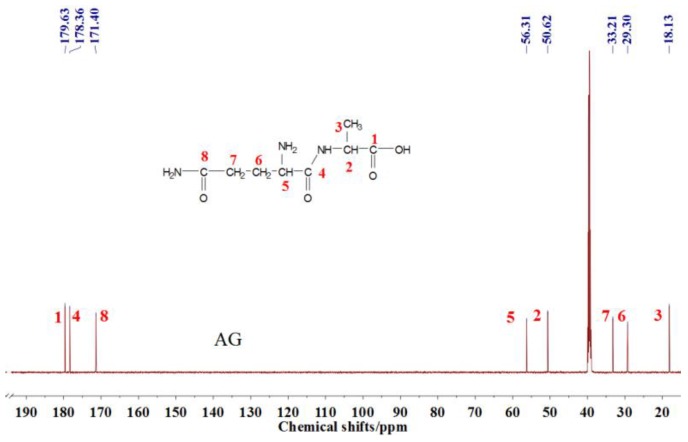
^13^C NMR spectra of *N*-(2)-l-alanyl-l-glutamine.

The ^13^C NMR result of AG is given in Figure 2. The clarity of the ^13^C NMR results of the AG sample reflected the purity of the raw material used in this study. All the carbons in an AG molecule and their assignments were labeled in Figure 2. Seen from the chemical structure of AG, both amino and carboxyl groups of AG molecules are possible to react with furfuryl alcohol molecules. When considering the unpaired electron of the amino group, the amino groups will show better nucleophilicity than carboxyl groups and be more liable to react with furfuryl alcohol molecules.

**Figure 3:**
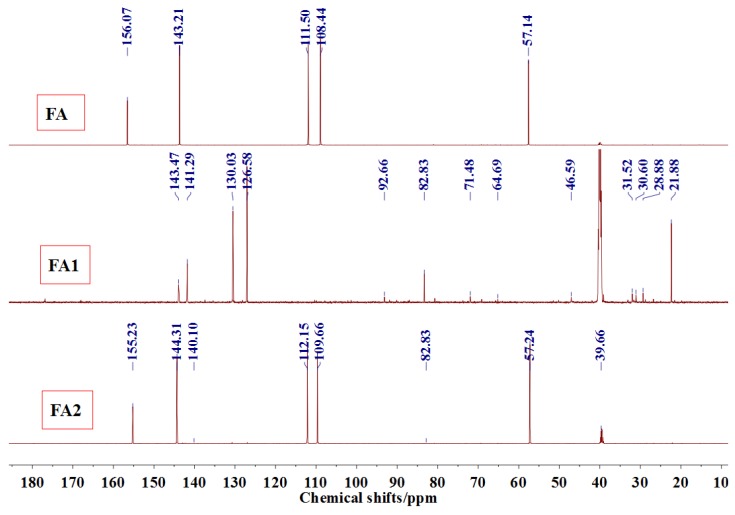
^13^C NMR spectra of furfuryl alcohol and the samples FA1 and FA2.

Figure 3 was the ^13^C NMR results of the material furfuryl alcohol (FA) and the samples FA1 and FA2. FA1, and FA2 were the products gotten with furfuryl alcohol under strong acid and alkaline conditions, respectively. The ^13^C NMR result of FA2 showed no obvious difference as that of FA, which indicated that there was no self-condensation between furfuryl alcohol molecules under strong alkaline conditions. On the contrary, the ^13^C NMR result of FA1 showed great difference as that of FA. In the ^13^C NMR of FA1, 28.88 and 31.52 ppm could be assigned as the methylene link from the self-condensation of furfuryl alcohol molecules. The shift at 64.69 ppm could be assigned as the methylene ether link, which came from the self-condensation of furfuryl alcohol, too. Since the peak area of methylene link was obviously bigger than that of ether link, methylene link might be the main form for the self-condensation of furfuryl alcohol under pH 1. In all, the ^13^C NMR proved that the self-condensation reaction of furfuryl alcohol under strong acid conditions. The shift at 82.83 ppm was assigned as the formaldehyde hydrate, namely methylene glycol, which indicated that the hydroxymethyl groups might be released from the furfuryl alcohol molecules or its self-condensation products under strong acid conditions. That also caused the appearance of the shift at 46.59, 71.48, and 92.66 ppm, which all came for formaldehyde and its derivatives.

**Figure 4:**
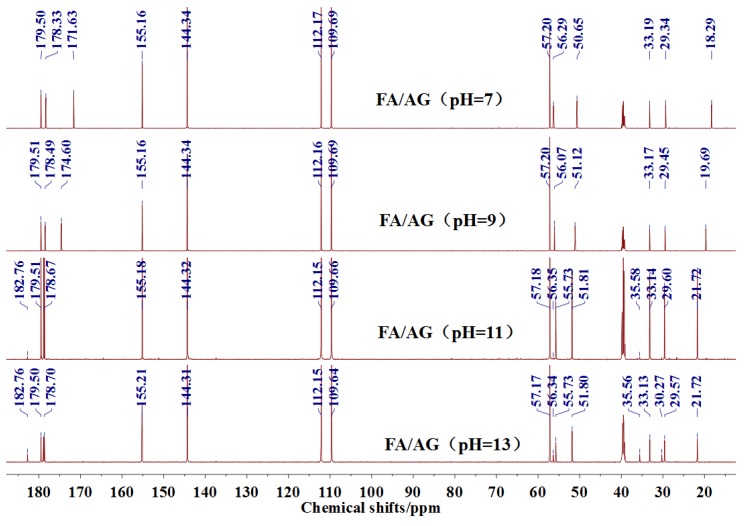
^13^C NMR spectra of the samples with furfuryl alcohol and *N*-(2)-l-alanyl-l-glutamine prepared under alkaline conditions.

The ^13^C NMR results of the samples prepared with furfuryl alcohol and AG under alkaline conditions were given as Figure 4.

Seen from Figure 4, the ^13^C NMR results of the samples that were prepared with furfuryl alcohol and AG under pH 7 and 9 showed no much difference from that of furfuryl alcohol and AG themselves, meaning no reaction between furfuryl alcohol and AG under pH 7 and 9. For the FA/AG samples that were prepared under pH 11 and 13, new peaks around 35.6 ppm appeared. To determine the assignment of this new peak, the software of Mestrenove was used to predict the chemical shifts of all the possible products gotten with furfuryl alcohol and AG. The chemical shift was actually determined greatly by the inductivity of the aliphatic amino, primary and secondary amido groups from AG molecules. The calculation of the software Mestrenove on the ^13^C NMR chemical shifts of the possible reactions between AG and furfuryl alcohol was then the results of respecting this law. According to the calculation results, the possible chemical shift of Furan–CH_2_–NH(CO)–R from the reaction between the primary amido group of AG and furfuryl alcohol was 36–37 ppm. The possible chemical shift of R–NH–CH_2_–Furan from the reaction between the aliphatic amine group of AG and furfuryl alcohol was 43–44 ppm. The possible chemical shift of Furan–CH_2_–NR’(CO)–R from the reaction between secondary amido group of AG and furfuryl alcohol was 40–41 ppm. Therefore, the new peak at 35.58 ppm for the sample prepared at pH 11 and 35.56 ppm for the sample prepared at pH 13 could be assigned as the Furan–CH_2_–NH(CO)–R structure from the reaction between the primary amido group of AG and furfuryl alcohol. The peak at 57.20 ppm could be assigned as –CH_2_ bond of furfuryl alcohol, which meant the existance of the free furfuryl alcohol in the system. It is normal and reasonable for the existence of the reaction monomer.

There was a peak at 182.76 ppm (pH = 11 and 13). Seen from the structure of AG, the self-condensation of intramolecular cyclisation of AG between carboxylic acid and primary amide could be occurred. However, the peak at 182.76 ppm should not be from carbonyl of the self-condensate of AG. When compared with the Figure 2 of the ^13^C NMR spectra of AG, 182.76 ppm appeared at a relatively lower field. In theory, if the self-condensation of intramolecular cyclisation of AG occurred, the carbonyl of the self-condensate would appeared at a relatively higher field because of the p–π conjugated effect in the new formed –CO–NH– groups. Similary, the peak at 182.76 ppm should not come from the C1 reacted with methylol groups of FA for the p–π conjugated effect.

There was a great possibility for the peak at 182.76 ppm from the shift of C1 of AG that was caused by reaction between C8 of AG and FA. Their reaction mechanism was similar with that between urea and formaldehyde under alkaline conditions.

However, for the ^13^C NMR, the chemical shift of carbonyl groups would be much easier to be affected by its environments, such as the resolvent, pH, and others, than that of methylene groups. The judgements on the reaction based on the analysis of the peaks of methylene groups would be more reliable than that of carbonyls groups.

There was no peak from methylene and ether link caused by the self-condensation of furfuryl alcohol molecules in Figure 4.

The analysis on Figure 4 indicated that the reaction between furfuryl alcohol and AG was difficult to happen under alkaline conditions and only when the alkaline condition was as strong as pH = 11 or higher, were a few co-condensation products that were detected in a mixing system with furfuryl alcohol and AG. The stability of furfuryl alcohol under alkaline conditions might be responsible for that [17,24]. Under relatively weak alkaline conditions, it seemed to be difficult for AG molecule to form a relatively stable primary amide anions reaction intermediate or even there were some amide anions in the system, their concentration was not as high as to promote the condensation reaction with furfuryl alcohol. Under strong alkaline conditions, to form more amide anions would get easier, which would be helpful for their combination with furfuryl alcohol. For the aliphatic amino groups of AG, it was hard to form reaction intermediate. For the secondary amido groups, the steric hindrance might be the main reason for the stop of condensation between them and furfuryl alcohol molecules.

Based on the analysis above, the reaction mechanism between furfuryl alcohol and AG was presumed, as below.


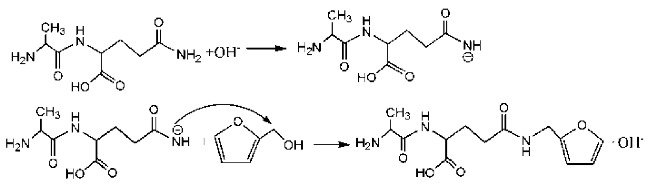


In all, under alkaline conditions, the condensation reaction between FA and AG was slow.

**Figure 5:**
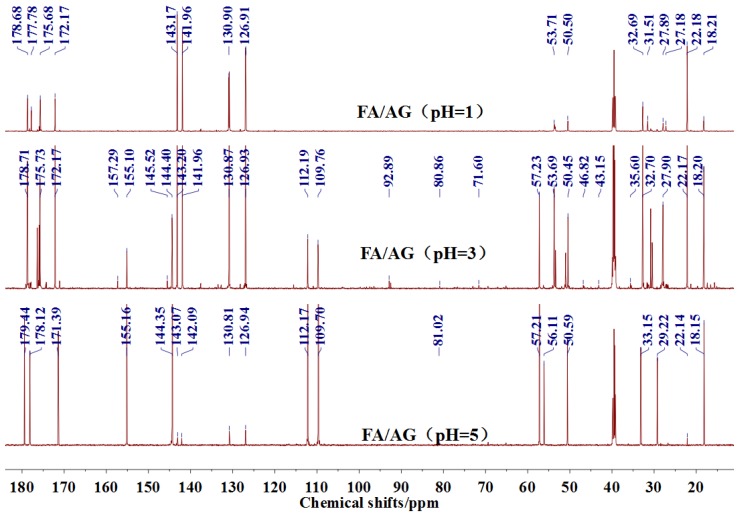
^13^C NMR spectra of the samples with furfuryl alcohol and *N*-(2)-l-alanyl-l-glutamine prepared under acid conditions

The ^13^C NMR results of the samples prepared with furfuryl alcohol and AG under pH 1, 3, and 5 were given as Figure 5.

In the mixing system with furfuryl alcohol and AG, the reaction intermediate might come from the hydroxymethyl furfural carbonium ions. They could react with its carbon 5, its hydroxymethyl groups or the amino groups of AG molecules. As seen from Figure 5, in the samples that were prepared with furfuryl alcohol and AG under pH 3, the peaks at 35.60 and 43.15 ppm were obviously observed. According to the analysis on the assignments of the chemical shift above, they could be assigned as Furan–CH_2_–NH(CO)–R and R–NH–CH_2_–Furan, respectively. Being similar with the results of the samples that were prepared under alkaline conditions, as seen from the bigger peak area of the former structure than that of the latter, the reaction between the primary amido groups of AG and furfuryl alcohol would be easier to proceed than that between the aliphatic amino groups of AG and furfuryl alcohol.

In theory, the amido groups in AG moleculs show weaker nucleophilicity than aliphatic amide groups because of the p–π conjugated effect of the amido groups. However, under acid conditions, aliphatic amide has the priority to attract hydrogen ions, and then be inactivated to other groups [23]. Therefore, under acid conditions, when compared with the aliphatic amide groups, the non-protonized amido groups would show bigger possibility to react with the furfural carbonium ions to form the Furan–CH_2_–NH(CO)–R structure. Being as the same as the results of the samples tha were prepared under alkaline conditions, no peak at around 41 ppm from the structure of Furan–CH_2_–NR’(CO)–R was detected.

The possible reactions with furfuryl alcohol and AG under acid conditions were as below.


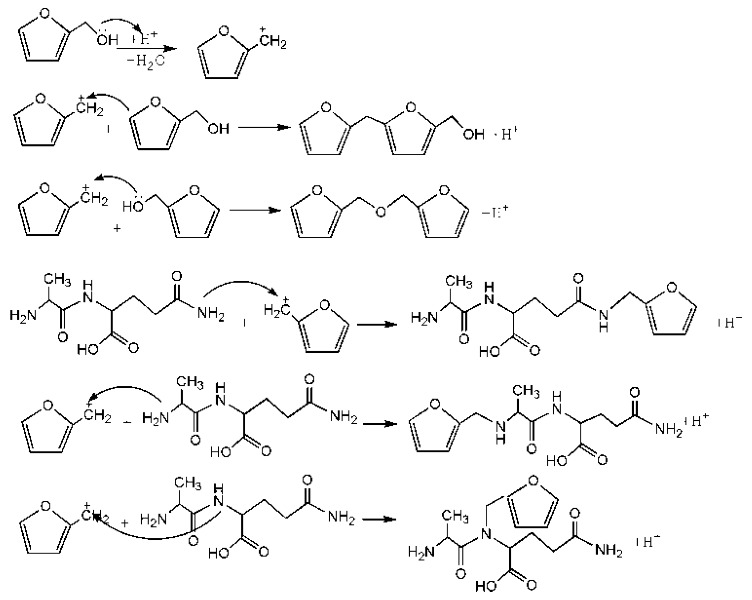


Seen from Figure 5, there were both self- and co-condensation products in the mixing system with furfuryl alcohol and AG under acid conditions, and the sample prepared under pH 3 showed the biggest amount of the co-condensation reaction products. Obviously, the co-condensation reaction between furfuryl alcohol and AG had to compete with the self-condensation of furfuryl alcohol. It was reported that violent self-condensation reaction between furfuryl alcohol molecules would happen under strong acid conditions, especially when the value of pH was lower than 2, and large quantities of heat would be given off [25,26]. The strong acid conditions would catalyze the reaction between the hydroxymethyl furfural carbonium ions and the furfuryl alcohol’s carbon 5. The release of the large quantities of heat meant that the self-condensation products of furfuryl alcohol molecules owned relatively low energy in the opinion of thermodynamics.

Both the relative higher energy of the co-condensation products of furfuryl alcohol and AG, and the possible protonation would lead to the prevailing of the self-condensation reaction in the mixing system with furfuryl alcohol and AG under acid conditions. The more acid the system was, the more violent the self-condensation would be. However, at the same time, with more acid in the mixing system, there would be more hydroxymethyl furfural carbonium ions, which would be favorable to the co-condensation reaction between furfuryl alcohol and AG. Therefore, there was an equilibrium pH for the competition between the self-condensation and co-condensation in the mixing system. In this study, for more co-condensation products, it was better for furfuryl alcohol and AG to be mixed under pH 3.

### 3.3. FT-IR Analysis

**Figure 6:**
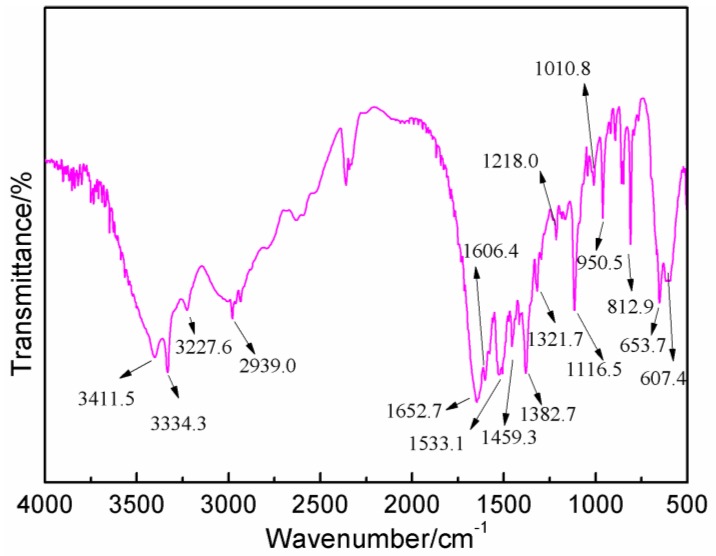
FT-IR spectrum of *N*-(2)-l-alanyl-l-glutamine.

**Figure 7:**
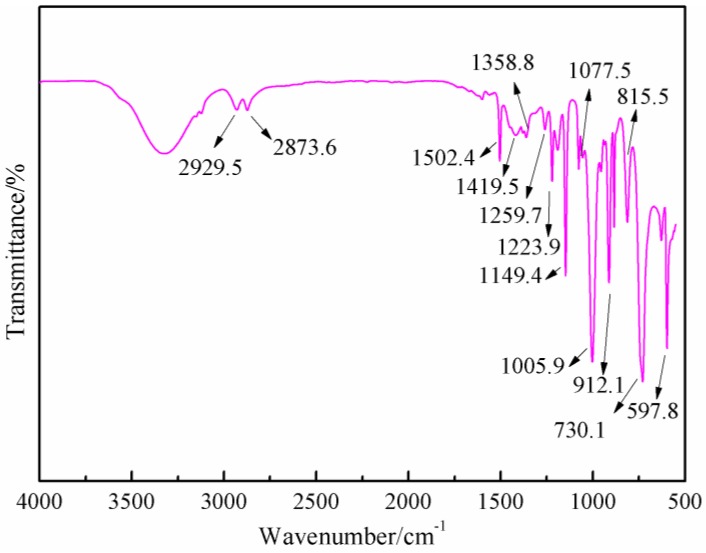
FT-IR spectrum of furfuryl alcohol (FA).

Figure 6 gives the FT-IR results of AG. In Figure 6, the absorption at 3334.3 and 3411.5 cm^−1^ could be assigned as the symmetric and asymmetric vibration of N–H bond of –NH_2_ groups. The absorption at 3227.6 cm^−1^ came from the vibration of N–H bond from –NH– groups. The absorption at 1652.7 cm^−1^ was from the C=O from amido groups. The absorption at 1606.4 cm^−1^ was from the bending vibration of N–H bond from aliphatic amino groups. The absorption at 2939.0 and 1321 cm^−1^ were, respectively, from O–H bond and C–O bond from carboxyl groups [27,28]. Figure 7 was the FT-IR results of furfuryl alcohol. The absorption at 3200 to 3300 cm^−1^ was from the vibration of hydroxyl from –CH_2_OH groups. The absorption at 2873.8 and 2929.5 cm^−1^ could be assigned as the symmetric and asymmetric vibration of methylene bond from –CH_2_OH groups. The absorption at 1005.9 cm^−1^ was from C–O of methylol groups. The absorption at 815.5 cm^−1^ was from the vibration of C-H of furan ring.

**Figure 8:**
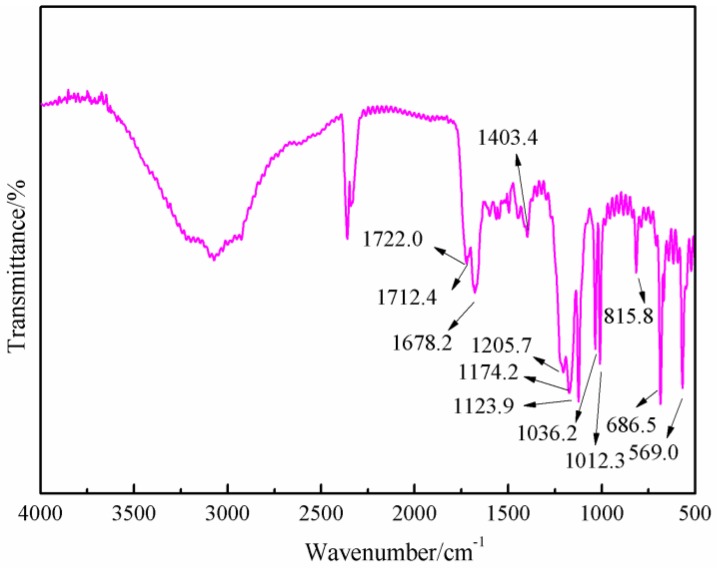
FT-IR spectrum of FA/AG prepared at pH = 3.

Figure 8 was the FT-IR results of the sample synthesized with furfuryl alcohol and AG under acid conditions, specifically at pH = 3. When compared with Figure 6, the absorption at 1652.7 and 1606.4 cm^−1^ from amido and aliphatic amino groups shifted, respectively, to 1722.0 and 1678.2 cm^−1^, which indicated that the combination of the amido and aliphatic amino groups with some groups with stronger inductivity than that of hydrogen atom. The absorption of 1420.4 cm^−1^ in Figure 6 from the vibration of C–N groups shifted to 1404.3 cm^−1^ in Figure 8, which indicated that the possible new group combined with the amido groups would weaken the p–π conjugated effect between amino and carbonyl bond. New absorptions appeared at 1205 and 1174.2 cm^−1^, which could be assigned as the symmetric and asymmetric vibration of methylene bond from –NH–CH_2_–C=C– groups. In all, the FT-IR results were another proof of the reaction between furfuryl alcohol and AG.

## 4. Conclusions

In this paper, to guide the preparation of soy protein-based adhesive, the reaction between protein and its cross-linker furfuryl alcohol were studied. To simplify the study, a simple dipeptide AG was used as a model compound of protein.

Based on the analysis on the results of ESI-MS, ^13^C NMR, and FT-IR of the samples that were prepared with furfuryl alcohol and AG under different pHs, it was found that the medium environment had great effects on the competition of the co-condensation reaction between furfuryl alcohol and AG and self-condensation reaction of furfuryl alcohol moleculs in the mixing system with furfuryl alcohol and AG. Under alkaline conditions, both co- and self-condensation were not obviously detected. Only when the value of pH was higher than 11, were a few co-condensation reaction products gotten. The reaction occurred mainly between furfuryl alcohol and the primary amido groups of AG. Under acid conditions, both co- and self-condensation were observed. When furfuryl alcohol and AG were mixed under pH 1, the self-condensation of furfuryl alcohol molecules prevailed over the co-condensation between furfuryl alcohol and AG. When furfuryl alcohol and AG were mixed under pH 5, both co- and self-condensation were not outstanding. In this study, under pH 3, the co- and self-condensation found equilibrium. There was a great possibility for the primary amido and aliphatic amino groups of AG molecules to react with furfuryl alcohol molecules. No reaction was detected between the secondary amido groups of AG and furfuryl alcohol.

## Figures and Tables

**Table 1:** The main ion peaks of ESI-MS and their assignments for the samples with furfuryl alcohol and *N*-(2)-l-alanyl-l-glutamine prepared under acidic conditions and their assignments.

Experimental (Da)		Chemical Species
[M + H]^+^	[M + Na]^+^	
218		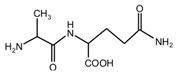
252		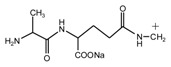
	293	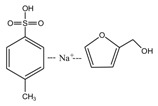
298		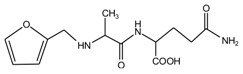
310		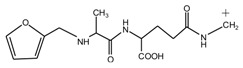
328		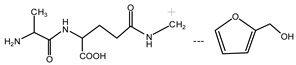
	338	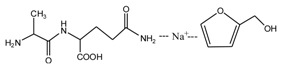
470		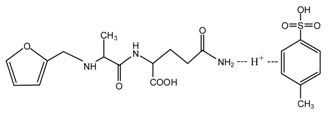
	533	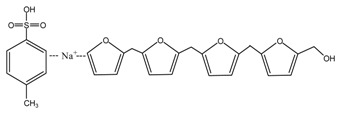
	617	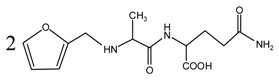

**Table 2:** The main ion peaks of ESI-MS and their assignments for the samples with furfuryl alcohol and *N*-(2)-l-alanyl-l-glutamine prepared under alkaline conditions and their assignments.

Experimental (Da)			Chemical Species
[M + H]^+^	[M + Na]^+^	[M + K]^+^	
218	240	256	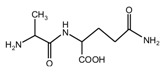 (AG)
	262		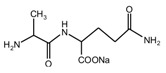 (AG-Na)
	299		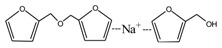
		315	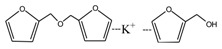
	318 (317)		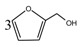
	397		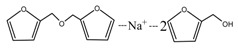
		413	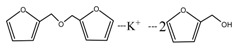
435			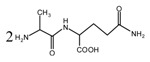
457			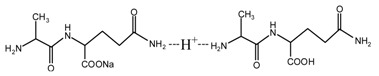
479			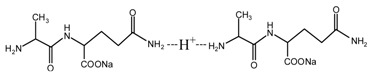
	501		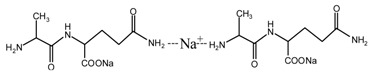
	718		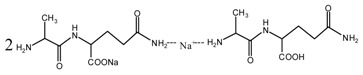
	740